# Novel Derivatives of Deoxycholic Acid Bearing Linear Aliphatic Diamine and Aminoalcohol Moieties and their Cyclic Analogs at the C3 Position: Synthesis and Evaluation of Their In Vitro Antitumor Potential

**DOI:** 10.3390/molecules24142644

**Published:** 2019-07-21

**Authors:** Andrey V. Markov, Valeriya O. Babich, Irina I. Popadyuk, Oksana V. Salomatina, Evgeniya B. Logashenko, Nariman F. Salakhutdinov, Marina A. Zenkova

**Affiliations:** 1 Institute of Chemical Biology and Fundamental Medicine, Siberian Branch of the Russian Academy of Sciences, Lavrent’ev ave., 8, 630090 Novosibirsk, Russia; 2 Novosibirsk State University, Pirogova str. 2, 630090 Novosibirsk, Russia; 3 N.N. Vorozhtsov Novosibirsk Institute of Organic Chemistry, Siberian Branch of the Russian Academy of Sciences, Lavrent’ev ave., 9, 630090 Novosibirsk, Russia

**Keywords:** deoxycholic acid derivatives, diamines, aminoalcohols, apoptosis, autophagy, VDR

## Abstract

A series of novel deoxycholic acid (DCA) derivatives containing aliphatic diamine and aminoalcohol or morpholine moieties at the C3 position were synthesized by 3,26-epoxide ring-opening reactions. These compounds were investigated for their cytotoxicity in four human tumor cell lines and murine macrophages and for inhibitory activity against macrophage-mediated NO synthesis in vitro. Obtained data revealed that: (i) all amine-containing substituents significantly increased the cytotoxicity of the novel compounds (IC_50_**^2–10^** = 1.0–36.0 μM) in comparison with DCA (IC_50_**^DCA^** ≥ 82.9 μM); (ii) aminoalcohol moieties were more preferable than diamine moieties due to the fact they imparted better selectivity for tumor cells of the novel derivatives; (iii) the susceptibility of tested cell lines to derivatives diminished in the following order: HuTu-80 (duodenal carcinoma) ≈ HepG2 (hepatocarcinoma) > KB-3-1 (cervical carcinoma) > RAW264.7 (macrophages) > A549 (lung carcinoma); (iv) compounds **8** and **9**, bearing aminoethanol and aminopropanol moieties, respectively, exhibited high cytotoxic selectivity indexes (SI^HuTu-80^ = 7.9 and 8.3, respectively) and good drug-likeness parameters; (v) the novel compounds do not display anti-NO activity. Mechanistic study revealed that compound **9** induces ROS-dependent cell death by activation of intrinsic caspase-dependent apoptosis and cytodestructive autophagy in HuTu-80 cells and vitamin D receptor can be considered as its primary target.

## 1. Introduction

Bile acids (BAs)—steroidal molecules synthesized in the liver of mammals—are essential for the absorption of dietary lipids and liposoluble vitamins. In a view of the fact that BAs, as well as other natural metabolites, display a wide spectrum of bioactivities based on their multitarget mode of action, these compounds have been the subject of numerous pharmacological studies [1,2]. In the past decades, intensive endeavors have been dedicated by investigators to design and synthesize semisynthetic BA derivatives characterized by improved antitumor potential and selectivity of action [3,4,5].

Diamine moieties are known to be considerable structural components of a multitude of bioactive compounds [6]. It was shown that introduction of such substituents in the steroid skeleton significantly increases the bioactivity level in comparison with the parent molecules, including antiallergic [7], antiproliferative [8,9], antifungal [9] as well as enzyme and BA transporter inhibitory [10,11,12,13,14,15] activities. Along with the diamine substituents, the nitrogen-containing moieties with other heteroatoms are also considered as attractive connecting units—the BA derivatives bearing them display diverse pharmacological effects. For instance, BA sulphonamides and cholic acid derivatives, containing amino acid or triazolyl fragments, show pronounced carbonic anhydrase inhibitory and anti-tuberculosis activities, respectively [16,17]. The particular attention of researchers in this field is also attracted to aminoalcohol moieties—it was shown that introduction of such fragments in BA molecules enhanced antifungal and lipid reducing activities [18,19] and improved gel-forming properties [20]. However, to the best of our knowledge, the anti-proliferative effects of aminoalcohol-containing BA derivatives have not been yet investigated.

Previously, we have synthesized a range of aliphatic diamine-bearing derivatives of deoxycholic acid (DCA) and analyzed their cytotoxicity with respect to a range of cultured human carcinoma cells [21]. In continuation of our investigations, we report herein the synthesis of novel derivatives of DCA substituted at C3 with aliphatic aminoalcohol and morpholine moieties and their anti-proliferative and anti-NO activities in vitro. In this paper, we also questioned how the replacement of the terminal amino group by oxygen-containing group in diamine substituents of DCA derivatives affected their biological properties and evaluated the mechanism of cell death-inducing activity of the leader compound **9**, bearing an aminopropanol moiety.

## 2. Results and Discussion

### 2.1. Synthesis

**Scheme 1:**
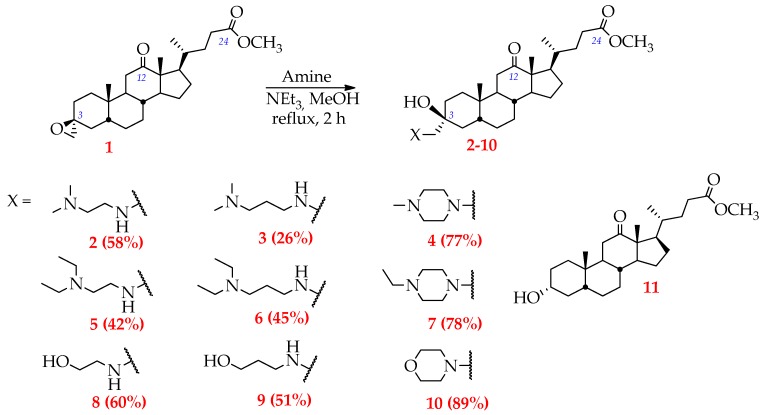
Reaction of epoxide **1** with different aliphatic amines containing an additional functional group—tertiary amine, hydroxyl and ether. 3α-hydroxy-12-oxo-5β-cholan-24-oate (**11**) was used for SAR analysis as a compound lacking amino-containing moieties at C3 position.

The present study is a continuation of our work on the transformation of DCA [21,22,23]. Here DCA was modified at the C3 position with a range of aliphatic amines containing an additional functional group—hydroxyl and ether (Scheme 1).

Compound **1** (methyl 3β-oxirane-12-oxo-5β-cholan-24-oate) was selected by us as a key intermediate due to the presence of epoxide ring strain, which significantly simplifies the introduction of new functional groups into the starting molecule by ring-opening reactions with a diversity of nucleophiles or electrophiles. This derivative was obtained according to a previously reported synthesis [23].

In our recent report [21], a range of aliphatic diamines (N,N-dimethylethylenediamine, N,N-diethylethylenediamine, N,N-dimethyl-1,3-propanediamine, N,N-diethyl-1,3-propanediamine, 1-methylpiperazine, 1-ethylpiperazine) were used by us as ring-opening agents. Obtained DCA derivatives bearing corresponding diamine substituents at the C3 position (compounds **2**–**7**) were evaluated for cytotoxicity in a range of human carcinoma cells [21]. Herein, in order to analyze the influence of the replacement of the terminal amino groups within the diamine moieties by oxygen-containing groups (hydroxyl and ether) on bioactivity of DCA derivatives, 2-aminoethanol, 3-aminopropanol and morpholine were taken for synthesis (compounds **8**–**10**).

The synthesis of novel DCA derivatives was carried out as depicted in Scheme 1. Epoxide ring-opening reactions with aminoalcohols and morpholine were performed according to the previously described method [21] and led to formation of single products (compounds **8**–**10**, Scheme 1) in yields ranging from 51% to 89% after purification by flash column chromatography. It should be noted that the yields of crude products **2**–**10** ranged from 80% to quantitative, however, the isolation and purification of the target compounds bearing aliphatic diamines or aminoalcohols by flash column chromatography was accompanied by a large loss of material. In this connection, only the syntheses of derivatives **4**, **7** and **10** containing at C3 substituted piperazines and morpholine can be viewed as suitable for scaling.

The ^1^H-NMR spectra of compounds **2**–**7** have been already described [21]. For compounds **8**–**10** characteristic ^1^H-NMR signals at *δ* 2.37 (for **8**), at *δ* 2.54 (for **9**) and at *δ* 2.24 (for **10**) were assigned to the C-3α methylene protons (CH_2_-26); the epoxide protons of **1** are located at *δ* 2.57 and 2.59 ppm [23]. Epoxide carbon signals are located at *δ* 52.85 ppm for C-26 and at *δ* 58.05 ppm for C-3 (^13^C-NMR data) [23]. After the epoxide ring-opening reaction signal shifts were observed: the signals of C-26 for **8, 9, 10** are at *δ* 61.51, 60.75, 69.52, respectively; signals of C-3 for **8**, **9**, **10** are at *δ* 70.33, 70.58, 70.51 ppm, respectively.

Thus, the suggested method permits the modification of the C3 position of bile acid scaffold, notably to introduce the novel functional groups in α-position instead of the native hydroxyl group with simultaneous forming of β-hydroxyl group. Additionally, for the SAR analysis, we used compound **11** (methyl 3α-hydroxy-12-oxo-5β-cholan-24-oate) containing only a hydroxy group at C3 (Scheme 1), that was synthesized according to a previously reported procedure [23].

### 2.2. Biological Evaluation

#### 2.2.1. Cytotoxicity of Novel DCA Derivatives and SAR Analysis

According to our previous data and data published by other research groups, chemical transformations of bile acids can greatly improve their antitumor potential [23,24,25]. Thus, as the first step of evaluation of the biological activities of our novel DCA derivatives we assessed their effects on tumor cell viability. The cytotoxicity of the compounds was investigated with respect to a panel of cultured cell lines, including human duodenal HuTu-80, hepatocellular HepG2, cervical KB-3-1 and lung A549 carcinomas as well as murine macrophage RAW264.7 and non-transformed human fibroblasts hFF3 by MTT assay. The cells were treated by the compounds in concentrations ranging from 1 µM to 100 µM for 24 h following by IC_50_ value calculations (Table 1). Compound **10** was found to be poorly soluble in cell culture medium; therefore, its maximal tested concentration was limited by 50 µM.

The part of obtained results on cytotoxicity of diamine-bearing DCA derivatives **2**–**7** have already been published by us (Table 1, compounds and IC_50_ values marked by asterisk) [21]. In the present work, we continued our research and supplemented these results with novel data obtained for aliphatic aminoalcohol- and morpholine-bearing compounds **8**–**10** and analyzed the effect of the replacement by hydroxyl or ester groups in the terminal amino group in the side moieties on the bioactivity of derivatives. As shown in Table 1, all modifications introduced at C3 significantly increased cytotoxicity of the novel compounds in comparison with parent molecule DCA (IC_50_**^2–10^** = 1.0–36.0 µM versus IC_50_**^DCA^** ≥ 82.9 µM). Moreover, novel derivatives are more cytotoxic than epoxide-bearing compound **1** (IC_50_^1^ ≥ 17.0 µM), which is a key intermediate in their synthesis. Analysis showed that amine-containing moieties at C3 position *per se*, but not the bile acid scaffold, underlie the high bioactivity of the derivatives—compound **11**, bearing only hydroxyl group at this position, displays significantly higher IC_50_ values (IC_50_**^11^** ≥ 25.7 µM). Duodenal HuTu-80 and liver HepG2 carcinoma cell lines were identified as the most susceptible to the novel derivatives, which can be explain by the peculiarities of bile acid metabolism, notably their enterohepatic circulation [26]. The obtained findings agreed well with the previously published data—we showed similar selective cytotoxicity with respect to cells of enterohepatic lineage for other DCA derivatives [22,23]. The most cytotoxic compound in the evaluated series of DCA derivatives was compound **6**, displaying the lowest IC_50_ values for almost all tested cell lines (IC_50_**^6^** = 1.0–6.8 µM).

SAR studies revealed a range of substitution patterns that improve the antitumor activity of DCA derivatives. We showed that:

(a) Replacement of terminal tertiary amino groups in the side moiety by the hydroxyl group is highly promising—derivatives **8** and **9**, containing aminoalcohol substituents, were found to display more pronounced selectivity for tested tumor cells in comparison with compounds, bearing corresponding diamine-containing group (cf., **8** to **2**/**5** and **9** to **3**/**6**);
(b) Elongation of the hydrocarbon chain between hydroxyl and amino groups has almost no effect on cytotoxicity (IC_50_**^8^** = 4.4–34.8 µM, IC_50_**^9^** = 4.0–33.3 µM), whereas, in the case of diamine-bearing derivatives, it was shown that a similar modification significantly increased the activity (IC_50_**^2^** = 7.1–36.0 µM and IC_50_**^3^** = 3.0–6.8 µM; IC_50_**^5^** = 3.3–25.5 µM and IC_50_**^6^** = 1.0–6.8 µM).
(c) Replacement of a hydroxyl group by an ether group, which limits the conformational flexibility, markedly reduces the cytotoxicity of derivatives with respect to non-malignant fibroblasts hFF3. Thus, compound **10**, bearing a morpholine substituent, is characterized by the highest IC_50_ values for this cell line (IC_50_^hFF3^ > 50 µM) among all tested DCA derivatives. However, this modification decreased the water solubility of the derivative and, therefore, limited its consideration as a lead compound for further studies.

**Figure 1:**
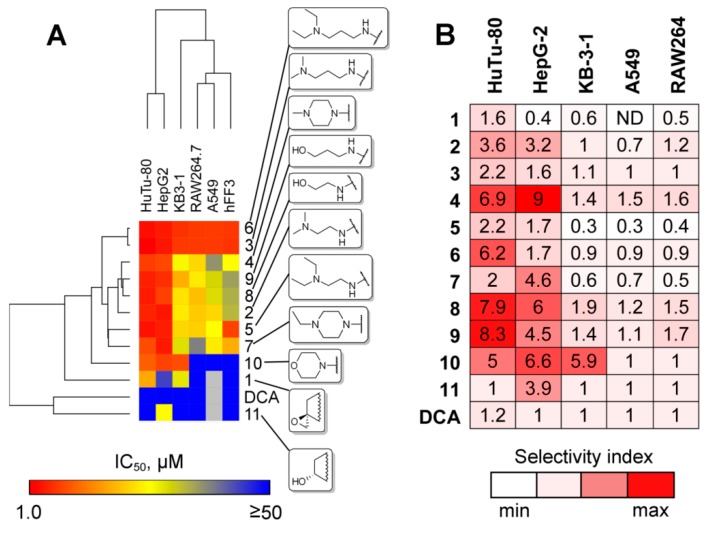
Cytotoxic profiles of novel DCA derivatives. (**A**) Hierarchical clustering of IC_50_ values of derivatives **1**–**11** and a parent compound DCA using Euclidean distance. (**B**) Heatmap showed selectivity of action of investigated compounds. SI was determined as the ratio of IC_50_ values in non-transformed fibroblasts to the IC_50_ values in the mentioned carcinoma/macrophage cells.

As the next step of our SAR analysis, we performed a hierarchical clustering of the obtained cytotoxic data in order to group the compounds according to the similarity of their cytotoxic profiles and reveal their cell context-dependent activity. As shown in the obtained cladogram, three major clades and one outgroup of investigated derivatives were identified (Figure 1A). The first clade includes compounds **3** and **6**, bearing diamine moieties with propane chains and displaying high toxicity against all tested cell lines. The second clade includes aminoalcohol-bearing compounds **8** and **9** and derivatives **2** and **4** containing the shortest diamine substituents in the tested series (Figure 1A). These compounds display marked cytotoxicity against enterohepatic tumor cell lines along with low effects on non-malignant fibroblast. Similar cytotoxic profiles were identified for compounds **5** and **7**, constituting the third clade, which are also characterized by enterohepatic mode of action, however, their toxicity to hFF3 fibroblasts is more pronounced (Figure 1A). Compound **10**, bearing morpholine moiety, forms the outgroup in obtained cladogram (Figure 1A)—it was shown that this derivative displays the high toxicity in enterohepatic and cervical tumor cells along with the minimal activity with respect to other tested cell lines. Compounds without amine-containing moieties (**1**, **11** and **DCA**) were found to lie separately in the cladogram and display moderate or low cytotoxicity in all tested cell lines. Performed clustering analysis clearly showed that aminoalcohol and short diamine substituents define high selectivity of derivatives’ action in enterohepatic tumor cells and can be considered as potent modifications. The increase of the relative lipophilicity of these derivatives is undesirable: (a) replacement of terminal methyl groups in a diamine moiety by ethyl groups significantly increases the cytotoxicity of compounds against non-malignant fibroblasts (compare **2** and **4** with **5** and **7**, respectively); (b) lengthening of carbon chain between two nitrogen atoms in the diamine moiety imparted cell context-independent cytotoxicity of derivative (compare **2** and **5** with **3** and **6**, respectively); (c) cyclization of aminoethanol side chain (**8**) to morpholine (**10**) decreases the solubility of derivatives in cell culture medium.

Clustering of cell lines according to their sensitivity to DCA derivatives revealed two main clades. The first one includes the most sensitive enterohepatic HuTu-80 and HepG2 cells; the second clade consists of residual tested cell lines, which are arranged in line with reduction of their sensitivity to investigated compounds: KB-3-1 > RAW264.7 > A549 > hFF3.

Next, in order to evaluate efficiency of antitumor effect of novel compounds, a Selectivity Index (SI) was defined as the ratio of IC_50_ for the derivatives in non-transformed fibroblasts versus IC_50_ in carcinoma cells. This revealed that the top-3 most active derivatives included aminoalcohol-bearing compounds **9** (SI = 8.3 and 4.5 in HuTu-80 and HepG2 cells) and **8** (SI = 7.9 and 6 in HuTu-80 and HepG2 cells) as well as methylpiperazine-containing derivative **4** (SI = 6.9 and 9 in HuTu-80 and HepG2 cells) (Figure 1B).

#### 2.2.2. Inhibitory Activity of Novel DCA Derivatives against NO Synthesis by Macrophages

Nitric oxide (NO) is an important intracellular and intercellular signaling molecule involved in regulation of a variety of physiological and pathophysiological processes, including vasodilation, immune response, inflammation and carcinogenesis. In the case of tumor progression, NO participated in regulation of aggressiveness of tumor growth, angiogenesis and metastasis [27]. In a view of revealed antitumor mode of action of novel DCA derivatives, it was interesting to determine whether these compounds affect NO production in vitro. Analysis showed that investigated derivatives does not significantly inhibit NO synthesis in IFNγ-stimulated RAW264.7 macrophages when used at non-toxic concentrations (IC_50_^NO^ > 10 μM) (Table 1).

#### 2.2.3. In silico ADME Analysis

For the next step of the study, we performed in silico ADME analysis of the novel DCA derivatives in order to evaluate their drug-like properties, namely how they conformed to Lipinski’s Rule of Five. According to Lipinski et al., an orally active drugs should satisfy the following properties: the molecule should not have more than: (a) five hydrogen bond donors, (b) ten hydrogen bond acceptors, (c) 500 Da molecular weight, and (d) an octanol-water partition coefficient log P value of 5 [28]. Using the SwissADME tool, we showed that only four of 11 investigated compounds fulfill all the mentioned conditions; the rest of the compounds violate Lipinski’s molecular weight value rule (Table 2).

As a result of the first part of the study, compounds **8** and **9** were identified as lead compounds displaying both the highest SI against tumor cells in the tested series and good drug-likeness parameters. Derivative **9**, demonstrating the highest cytotoxicity with respect to the most sensitive HuTu-80 cells, was chosen as a model compound to reveal the mechanism of the cytotoxic effects of these novel DCA derivatives on tumor cells.

#### 2.2.4. Mechanism of Cytotoxic Effect of Compound **9**

Semisynthetic bile acid derivatives are known to induce cell death mainly by activation of apoptosis [24]—an energy-dependent process of programmed cell death, characterized by a specific biomarker profile, including perturbations in transmembrane lipid asymmetry and mitochondrial homeostasis, activation of caspase cascade as well as DNA fragmentation [29]. Thus, in the mechanistic study we questioned whether compound **9** induced apoptosis in HuTu-80 cells. In order to understand this we analyzed its modulatory effects on the apoptosis-related processes mentioned above; moreover, the ability of compound **9** to trigger other cell death pathways, including necrosis, cell cycle arrest and autophagy, was investigated.

##### Compound **9** Induces Intrinsic Caspase-Dependent Apoptosis

In the first step of the mechanistic study, the effect of compound **9** on phosphatidylserine (PS) externalization, a marker of the early phase of apoptosis, was analyzed by double staining of compound **9**-treated HuTu-80 cells by annexin V FITC and propidium iodide (PI). Live cells are characterized by lipid membrane asymmetry—PS is mainly present in the inner leaflet of the plasma membrane, whereas phosphatidylcholine and sphingomielin are restricted to the outer leaflet. During apoptosis the activity of enzymes involved in membrane lipid scrambling is inhibited and PS is exposed on cell surface [30]. Annexin V is a protein that has a high affinity for PS and its conjugates with fluorophores can be used to estimate the level of PS externalization in vitro. Usage of PI gives in turn an opportunity to identify late apoptotic and necrotic cells displaying increased membrane permeability.

**Figure 2:**
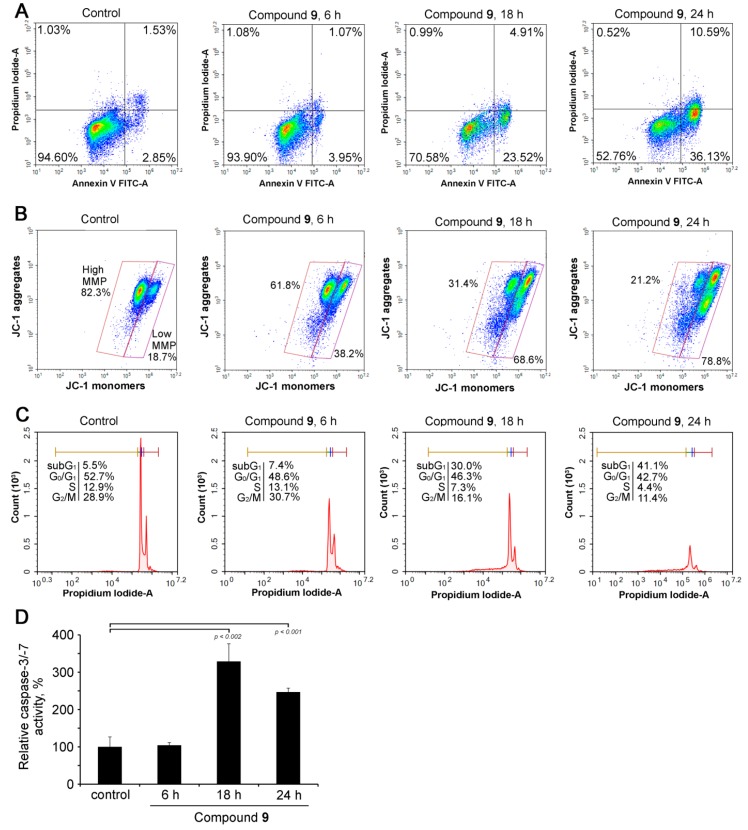
Compound **9** induces apoptosis in HuTu-80 cells. (**A**) Compound **9** caused PS externalization. HuTu-80 cells were treated by compound **9** at 8 µM for 24 h, stained by Annexin V FITC/PI and analyzed by flow cytometry. The percentage of live (lower left quadrant), early apoptotic (lower right quadrant), late apoptotic (upper right quadrant) and necrotic (upper left quadrant) cells were shown. (**B**). Compound **9** causes loss of mitochondrial membrane potential. HuTu-80 cells were treated by compound **9** at 8 µM for indicated times, stained with JC-1 and analyzed by flow cytometry. (**C**). Effect of compound **9** on cell cycle distributions in HuTu-80 cells. HuTu-80 cells were treated with compound **9** at 8 µM for indicated times, stained with PI and analyzed by flow cytometry. (**D**). Compound **9** acivates caspase-3/7 in HuTu-80 cells. HuTu-80 cells were treated by compound **9** at 8 µM for indicated times followed by incubation with a Caspase-Glo^®^ 3/7 Assay kit (Promega, Madison, WI, USA).

As shown in Figure 2A, compound **9** induced PS externalization in HuTu-80 cells in a time-dependent manner. Treatment of the cells by derivative **9** at 8 μM for 6 h did not cause early apoptotic changes—a significant similarity in cell populations in compound **9**-treated and control groups was observed (Figure 2A). Increase of treatment duration up to 18 h and 24 h caused marked accumulation of early apoptotic cells (Annexin V FITC+/PI-, right lower quadrant) from 2.9% (control) to 23.5% and 36.1%, respectively. Additionally, the percentage of late apoptotic population (Figure 2A, Annexin V FITC+/PI+, right upper quadrant) was also increased in compound **9**-treated group at 18 h and 24 h time points up to 4.9% and 10.6%, respectively, in comparison with the control (1.5%). We also revealed that compound **9** did not induce necrosis—the percentage of necrotic cells (Annexin V FITC-/PI+, left upper quadrant) were shown to be similar in the control and experimental cells and not exceed 1.1%.

Next, in order to identify whether the compound **9** induces apoptotic cell death by triggering mitochondrial dysfunction, we analyzed its effect on mitochondrial membrane potential (Δψ_m_) in HuTu-80 cells by using fluorescent cationic JC-1 dye (Figure 2B). JC-1 is characterized by potential-dependent accumulation in mitochondria—in normal condition the dye accumulates within this organelle and forms aggregates, exhibiting red fluorescence, whereas in apoptotic cells with collapsed Δψm JC-1 remains in cytoplasm as a monomer, displaying green fluorescence [31]. Using flow cytometry analysis, we revealed that compound **9** caused the loss of Δψ_m_ in HuTu-80 cells in a time-dependent manner—the percentage of the cells with collapsed membrane potential significantly increased from 18.7% in control group to 38.2%, 68.6% and 78.8% in HuTu-80 cells, treated by compound **9** for 6 h, 18 h and 24 h, respectively (Figure 2B). Obtained results clearly showed that investigated DCA derivative **9** induces cell death by activation of mitochondrial pathway of apoptosis.

It is known that the cytotoxic effects of bile acids and their derivatives can be determined not only by their pro-apoptogenic activity, but also by modulation of cell cycle distribution [24]. In order to clarify whether compound **9** induces cell cycle arrest in HuTu-80 cells, analysis of cell cycle by flow cytometry was performed. As shown in Figure 2C, treatment of HuTu-80 cells by compound **9** caused time-dependent accumulation of subdiploid cell population (subG_1_), corresponding to apoptotic cells with fragmented DNA, up to 7.4%, 30.0% and 41.1% for 6 h, 18 h and 24 h time points, respectively, with decreases in percentage of cells in G_0_/G_1_, S and G_2_/M phases (Figure 2C). Thus, performed analysis showed that compound **9** did not cause cell cycle arrest in the used treatment regimen and independently confirmed its pro-apoptogenic activity in HuTu-80 cells.

In the next step of the study our attention was focused on understanding the ability of compound **9** to activate caspases—a family of cysteine proteases, being critical mediators of apoptosis. In order to perform this, we evaluated the effect of compound **9** on activity of key executioner caspases-3 and -7 responsible for proteolytic cleavage of many proteins in the culminating step of cell death. Using conjugates of tetrapeptide DEVD, a caspase-3/7 substrate, with aminoluciferin, we showed that compound **9** significantly increased caspase-3/7 activity in HuTu-80 cells at 18 h and 24 h time points (Figure 2D). Taken together, obtained results clearly showed the ability of compound **9** to induce mitochondrial caspase-dependent apoptosis in HuTu-80 cells.

##### Compound **9** Triggers Cytodestructive Autophagy

It is known that bile acids can induce cell death independently of apoptosis by activation of autophagy [32,33]. Autophagy is a regulated lysosome-dependent pathway involved in the degradation of damaged and obsolete intracellular components [34]. During autophagy, these components are isolated by a bilayer membrane resulting in autophagosome formation. Autophagosomes than fuse with lysosomes that leads to enzymatic degradation of their contents [34]. During prolonged and strong stress, cells can autophagocytize up to a half of their cytoplasm that leads to a collapse of cell functions and, as a result, cell death [35]. Monodansylcadaverin (MDC) is an autofluorescent amine that specifically accumulates within autophagic vacuoles and, therefore, can be used as a marker of autophagy [36].

**Figure 3:**
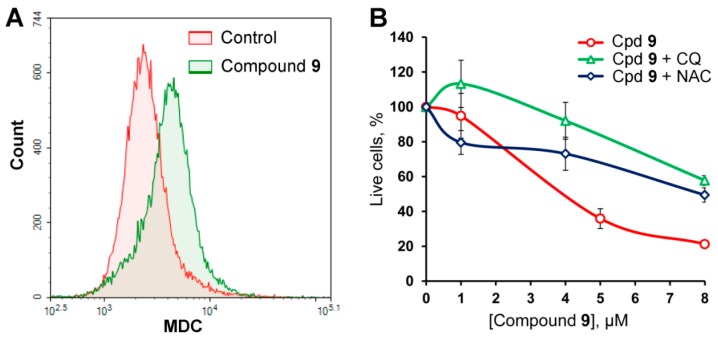
Compound **9** triggers cytodestructive autophagy in HuTu-80 cells. (**A**) HuTu-80 cells were treated by compound **9** at 8 µM for 24 h, stained by MDC and analyzed using flow cytometry. (**B**) The effect of autophagy inhibitor chloroquine (CQ) (50 µM) and ROS scavenger N-acetyl cysteine (NAC) (2 mM) on viability of HuTu-80 cells, treated by compound **9** (Cpd **9**) (8 µM) for 24 h.

In order to reveal whether compound **9** could induce autophagy, HuTu-80 cells were treated by the derivative at 8 μM for 24 h, stained by MDC and analyzed by flow cytometry. As shown in Figure 3A, the intensity of MDC-associated fluorescence in compound **9**-treated cells was significantly higher than in control that definitely showed the ability of compound **9** to trigger autophagy in HuTu-80 cells.

Autophagy is known to display both cytoprotective and cytodestructive effects depending on the level of cellular stress [35]. In view of this, we further investigated the role of autophagy in compound **9**-induced cell death. We showed that co-incubation of compound **9**-treated HuTu-80 cells with chloroquine (CQ), an autophagy inhibitor, significantly decreased the cytotoxicity of investigated derivative—the IC_50_ values of compound **9** alone or in the presence of CQ were found to be 4 μM and >8 μM, respectively (Figure 3B). Thus, obtained data revealed that compound **9** can induce cytodestructive autophagy equally with apoptosis in HuTu-80 cells.

As reactive oxygen species (ROS) are known to play an important role in regulation of apoptosis and autophagy [37,38], we further examined whether ROS production is involved in compound **9**-induced cell death. To understand this, HuTu-80 cell were pretreated with the free radical scavenger N-acetyl-L-cysteine (NAC) 1 h prior to the administration of compound **9** and then cell viability at 24 h was measured by MTT assay. As shown in Figure 3B, pretreatment with NAC significantly decreased cytotoxicity of compound **9**. These results therefore suggest that compound **9**-induced cell death of HuTu-80 cells is mediated by the production of ROS.

##### VDR is a Probable Primary Target of Compound **9**

In order to fully understand the molecular mechanism of action of novel bioactive compounds it is highly important to know their primary protein targets in the cells. Bile acids (BAs), like other natural compounds, are characterized by a multitarget mode of action [39]. Among all the diversity of BAs’ primary targets two classes of proteins were the most investigated, notably a family of nuclear hormone receptors, including farnesoid X receptor (FXR), pregnane X receptor (PXR), vitamin D receptor (VDR) and constitutive androstane receptor (CAR), as well as transmembrane G protein-coupled receptor TGR5 [40]. BAs can bind to and activate these receptors that alter the expression of numerous genes involved in regulation of cell metabolism and proliferation [40].

It was found that chemical transformations of BAs can increase their agonistic activity on mentioned receptors: Ishizawa with colleagues showed that introduction of short-chain fatty acids at C3 position of lithocholic acid (LCA) significantly enhanced its VDR stimulating activity, and elongation of the side moiety proportionally increased the VDR specificity of derivatives [41]. It was shown that LCA propionate can be considered as VDR-selective BA derivative, which activates VDR at concentrations that are not effective on other BA-sensible receptors [41].

Considering certain similarities in the structure of compound **9** with LCA propionate, notably the presence of long unbranched side chain at the C3 position of BA molecule, we supposed that VDR can be considered as probable primary target of compound **9**. Moreover, activation of VDR was shown to be able to induce apoptosis and autophagy [42,43,44] that correlates well with ability of compound **9** to trigger these processes. Particularly, a range of VDR ligands displays a pro-apoptogenic activity—an active metabolite of vitamin D, 1α,25(OH)_2_D_3_, was found to increase Bax/Bcl-2 ratio in leukemia cells [45] and trigger cytochrome c release from mitochondria with subsequent activation of caspase cascade in prostate cancer cells [46]. Apoptosis-inducing activity of VDR ligands was also shown for the Gemini vitamin D analogue of 1α,25(OH)_2_D_3_ [47] and diarylmethane skeleton-containing VDR agonist [48] in colon and breast cancer cells, respectively. Beside this, activation of VDR can stimulate autophagy—previously, it was found that 1α,25(OH)_2_D_3_ and calcipotriol induce autophagy in breast and colorectal cancer [44,49] and cervical carcinoma [50] cells, respectively.

**Figure 4:**
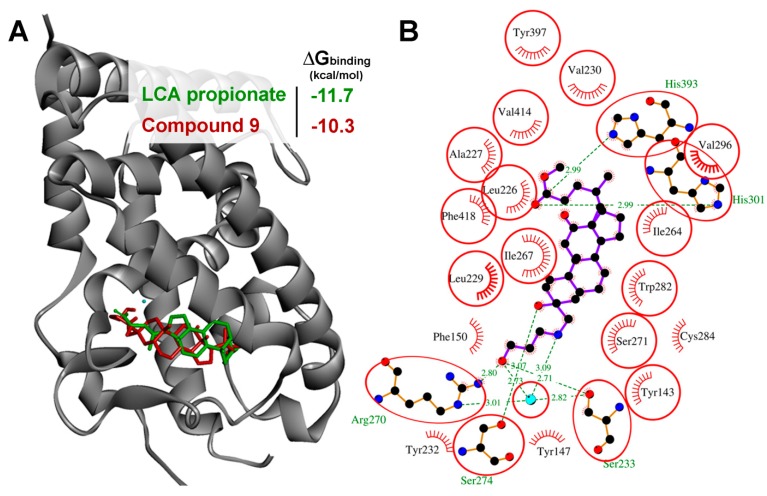
VDR is a probable primary target of compound **9**. (**A**) The mode of binding of compound **9** to VDR. Stereo presentation of docked pose of compound **9** in VDR, superimposed on LCA propionate-bound structure (PDB ID: 3W5T), was drown by BIOVIA Discovery Studio. Structures of LCA propionate and compound **9** are depicted in blue and red sticks, respectively. (**B**) 2D representation of docked pose of compound **9** in VDR is created by LigPlot+. The comb and green dashed lines represent hydrophobic interactions and hydrogen bonds, respectively. Common residues, interacting with both LCA propionate and compound **9**, are highlighted in red circles. Color coding of atoms in ball format: black—carbon, blue—nitrogen, red—oxygen, sky blue—water molecule.

In order to assess the ability of compound **9** to bind to VDR, molecular docking simulations were performed. Our results showed that compound **9** can snugly fit into the ligand-binding pocket of VDR in positions very close to that of LCA propionate (Figure 4). As shown in Figure 4B, the docking data suggested that binding of compound **9** to VDR involves 7 hydrogen bonds between the OH group at C3 position with the side chain of Ser274 (3.07 Å), the terminal OH group in aminopropanol moiety with Ser233, Arg270 and water molecule (2.71 Å, 2.80 Å and 2.73 Å, respectively) and the carbonyl group at C24 position with the backbones of His301 and His393 (2.99 Å). The steroid core of compound **9** is also stabilized by hydrophobic residues Tyr143, Tyr147, Phe150, Leu226, Ala227, Leu229, Val230, Ile264, Ile267, Ser271, Trp282, Cys284, Val296, Tyr397, Val414 and Phe418, the majority of which participate in the binding of LCA propionate (Figure 4B). Compound **9** was found to display a high binding affinity with very low binding energy (−10.3 kcal/mole) similar to that of LCA propionate (−11.7 kcal/mole). Thus, these data indicates that compound **9** can target VDR and probably causes its activation.

## 3. Materials and Methods

### 3.1. General Experimental Procedures and Reagents

Elemental analyses were performed on a EURO EA3000 automated CHNS-analyser (EuroVector, Milan, Italy). Analyses indicated by the symbols of the elements were within ±0.4% of theoretical values. Melting points were determined on a FP900 thermosystem (Mettler Toledo, Greifensee, Switzerland) and are uncorrected. The elemental composition of the products was determined from high-resolution mass spectra recorded on a double focusing sector (DFS) instrument (Thermo Electron Corporation, Bremen, Germany). Optical rotations were measured with a PolAAr 3005 polarimeter (Optical Activity Ltd., Huntingdon, UK). ^1^H- and ^13^C-NMR spectra were measured on an AV-600 spectrometer (operating frequency 600.30 MHz for ^1^H and 150.95 MHz for ^13^C; Bruker BioSpin GmbH, Rheinstetten, Germany). Solutions of each compound were prepared in CDCl_3_. Chemical shifts were recorded in δ (ppm) using δ 7.24 (^1^H- NMR) and δ 76.90 (^13^C-NMR) of CHCl_3_ as internal standards. Chemical shift measurements were given in ppm and the coupling constants (*J*) in Hertz (Hz). The structure of the compounds was determined by NMR using standard one-dimensional and two-dimensional procedures (^1^H-^1^H COSY, ^1^H-^13^C HMBC/HSQC, ^13^C-^1^H HETCOR/COLOC). The purity of the final compounds and intermediates for biological testing was >95% as determined by HPLC analysis. HPLC analyses were carried out on a MiLiChromA-02 system (EcoNova Ltd., Novosibirks,), using a ProntoSIL 120-5-C18 AQ column (2.0 × 75 mm column, particle size 5.0 μm, Bischoff, Leonberg, Germany). The mobile phase was Millipore purified water with 0.1% trifluoroacetic acid at a flow rate of 150 µL/min at 35 °C with UV detection at 210, 220, 240, 260 and 280 nm. A typical run time was 25 min with a linear gradient of 0–100% methanol. Flash column chromatography was performed with silica gel (60–200 mesh, Merck, Darmstadt, Germany). All course of all reactions were monitored by TLC analysis using Merck 60 F254 silica gel on aluminum sheets with the eluent CHCl_3_-MeOH (20:1,5 or 20:3), CHCl_3_-AcOEt (20:3) or CH_2_Cl_2_- MeOH (20:2).

DCA (99%) was purchased from abcr GmbH & Co. KG (Karlsruhe, Germany). N,N-Dimethylethylenediamine (99%), N,N-dimethyl-1,3-propanediamine, N,N-diethyl-1,3- propanediamine (99%), and 1-methylpiperazine were purchased from ACROS Organics (Geel, Belgium). N,N-Diethylethylenediamine (99%) and 1-ethylpiperazine (98%) were purchased from Sigma Aldrich (St. Louis, MO, USA). 3-Amino-1-propanol (99%) was purchased from Alfa Aesar (Ward Hill, MA, USA). Morpholine (99%) was purchased from Merck (Darmstadt, Germany). 2-Aminoethanol was purchased from Ekos-1 (Moscow, Russia). All solvents used in the reactions were purified and dried according to previously reported procedures.

### 3.2. General Procedure for the Synthesis of Compounds ***8**–**10***

Calculated amounts of epoxide **1** (1 equiv.), amine (1.5 equiv.) and 3–5 drops of NEt_3_ in methanol (10–25 mL) were refluxed for 2 h. Then the reaction mixture was evaporated and redissolved in CHCl_3_–Et_2_O (for compounds **8, 9**) or EtOAc (for compound **10**). Organic layers were washed with NaCl (aq.) and H_2_O, dried over anhydrous MgSO_4_ and evaporated to dryness.

#### 3.2.1. Methyl 3-hydroxy-3-((2-hydroxyethylamino)methyl)-12-oxo-5β-cholan-24-oate (**8**)

The crude product was obtained as an amorphous white solid (1.34 g, 97%) from epoxide **2** (1.2 g, 2.9 mmol) and 2-aminoethanol (0.26 mL, 4.3 mmol) according to the general procedure. The crude product was purified by flash column chromatography (silica gel, 0–20% MeOH in CHCl_3_) to yield compound **8** (0.83 g, 60%) as an amorphous white solid. Mp 154.3–158.4°C. $\left[\alpha_{D}^{23.7}\right]$ +90° (*c* 0.10, CHCl_3_). HRMS: *m/z* calcd for C_28_H_47_NO_5_: 477.3449; found: 477.3456. ^1^H-NMR (DMSO-d_6_): *δ* = 4.43 (br.s., OH), 3.88 (br.s., OH), 3.57 (s, 3H, CH_3_-25), 3.43 (m, 2H, CH_2_-28), 3.32 (s, NH), 2.55 (dd, 2H, *J = J* = 5.7, CH_2_-27), 2.48 (dd, 1H, ^2^*J = J_11a,9a_* = 12.7, H-11*a*(β)), 2.37 (s, 2H, CH_2_-26), 2.33 (m, 1H, H-23), 2.21 (m, 1H, H-23′), 1.90–1.57 (m: 10H, [1.85]-H-17, [1.85]-H-11*e*(α), [1.85]-H-6, [1.83]-H-16, [1.77]-H-8, [1.71]-H-22, [1.70]-H-5, [1.67]-H-9, [1.63]-H-15, [1.61]-H-4*), 1.45-1.37(m: 2H, [1.40]-H-1*, [1.40]-H-7), 1.34–0.99 (m: 11H, [1.29]-H-14, [1.29]-H-15′, [1.28]-H-1′*, [1.27]-H-16′, [1.26]-H-2*, [1.22]-H-22′, [1.22]-H-20, [1.18]-H-2′*, [1.12]-H-6′, [1.09]-H-4′*, [1.04]-H-7′), 0.98 (s, 3H, CH_3_-19), 0.96 (s, 3H, CH_3_-18), 0.74 (d, 3H, *J_21,20_* = 6.3, CH_3_-21). ^13^C-NMR (DMSO-d_6_): *δ* = 213.78 (s, C-12), 173.64 (s, C-24), 70.33 (s, C-3), 61.51 (t, C-26), 60.38 (t, C-28), 57.91 (d, C-14), 56.81 (s, C-13), 52.61 (t, C-27), 51.17 (q, C-25), 46.14 (d, C-17), 42.66 (d, C-9), 37.99 (t, C-11), 36.92 (d, C-5), 35.70 (t, C-4*), 35.38 (s, C-10), 34.97 (d, C-20), 34.90 (d, C-8), 31.09 (t, C-1*), 30.65 (t, C-23), 30.27 (t, C-22), 29.77 (t, C-2*), 27.00 (t, C-16), 26.42 (t, C-6), 25.56 (t, C-7), 23.78 (t, C-15), 22.90 (q, C-19), 18.56 (q, C-21), 11.27 (q, C-18) (The ^1^H- and ^13^C-NMR spectra of compound **8** and other synthesized DCA derivatives see in Supplementary Materials). Signal assignments marked with an asterisk are unreliable.

#### 3.2.2. Methyl 3-hydroxy-3-((3-hydroxypropylamino)methyl)-12-oxo-5β-cholan-24-oate (**9**)

The crude product was obtained as an amorphous white solid (1.34 g, 97%) from epoxide **2** (1.2 g, 2.9 mmol) and 3-amino-1-propanol (0.34 mL, 4.3 mmol) according to the general procedure. The crude product was purified by flash column chromatography (Al_2_O_3_, 0–100% CH_2_Cl_2_ in benzene, then 0–5% MeOH in CHCl_3_) to yield compound **7** (0.72 g, 51%) as an amorphous white solid. Mp 119.5 °C [decomposition]. $\alpha_{D}^{23.8}$ +84° (*c* 0.10, CHCl_3_). HRMS: *m/z* calcd for C_29_H_49_NO_5_: 491.3605; found: 491.3607. ^1^H-NMR (CDCl_3_): *δ* = 3.75 (dd, 2H, *J* = 5.5, CH_2_-29), 3.62 (s, 3H, CH_3_-25), 3.53 (br. s., OH), 2.86 (m, 2H, CH_2_-27), 2.54 (d, 1H, *J* = 14.0, H-26), 2.51 (d, 1H, *J* = 14.0, H-26′), 2.46 (dd, 1H, *J* = 12.7, H-11*a*(β)), 2.35 (m, 1H, H-23), 2.21 (m, 1H, H-23′), 2.02–1.94 (m: 2H, [1.97]-H-17, [1.97]-H-11*e*(α)), 1.93–1.83 (m: 2H, [1.89]-H-16, [1.87]-H-6), 1.82-1.60 (m: 7H, [1.78]-H-22, [1.77]-H-8, [1.76]-H-5, [1.72]-CH_2_-28, [1.67]-H-15, [1.65]-H-9), 1.56 (dd, 1H, *J* = 13.6, H-4), 1.51–1.15 (m: 12H, [1.46]-H-7, [1.43]-H-1, [1.36]-H-1′, [1.36]-H-2, [1.32]-H-22′, [1.31]-H-14, [1.28]-H-16′, [1.27]-H-15′, [1.26]-H-2′, [1.24]-H-20, [1.22]-H-4′, [1.20]-H-6′), 1.01 (s, 3H, CH_3_-19), 0.99 (m, 1H, H-7′), 0.98 (s, 3H, CH_3_-18), 0.80 (d, 3H, *J_21,20_* = 6.6, CH_3_-21). ^13^C-NMR (CDCl_3_): *δ* = 215.07 (s, C-12), 174.52 (s, C-24), 70.58 (s, C-3), 60.82 (t, C-29), 60.75 (t, C-26), 58.67 (d, C-14), 57.42 (s, C-13), 51.32 (q, C-25), 49.47 (t, C-27), 46.31 (d, C-17), 43.44 (d, C-9), 38.26 (t, C-11), 37.30 (d, C-5), 35.90 (t, C-4), 35.65 (s, C-10), 35.46 (d, C-20), 35.35 (d, C-8), 31.16 (t, C-1), 31.16 (t, C-23), 30.52 (t, C-28), 30.37 (t, C-22), 30.19 (t, C-2), 27.36 (t, C-16), 26.38 (t, C-6), 25.85 (t, C-7), 24.16 (t, C-15), 22.85 (q, C-19), 18.44 (q, C-21), 11.53 (q, C-18).

#### 3.2.3. Methyl 3-hydroxy-3-(morpholinomethyl)-12-oxo-5β-cholan-24-oate (**10**)

The crude product was obtained as a white solid (1.17 g, 98%) from epoxide **1** (1.0 g, 2.4 mmol) and morpholin (0.31 mL, 3.6 mmol) according to the general procedure. The crude product was purified by flash column chromatography (silica gel, CH_2_Cl_2_) to yield compound **10** (1.07 g, 89%) as a white solid. An analytically pure sample was obtained by re-crystallization from mixture AcOEt—*n*-hexane. Mp 141.8–144.8 °C. $\alpha_{D}^{29.9}$ +110° (*c* 0.10, CHCl_3_). HRMS: *m/z* calcd for C_30_H_49_NO_5_: 503.3605; found: 503.3596. ^1^H-NMR (CDCl_3_): *δ* = 3.65 (m, 4H, CH_2_-29, CH_2_-30), 3.61 (s, 3H, CH_3_-25), 2.58 (br.s., 4H, CH_2_-27, CH_2_-28), 2.45 (dd, 1H, *^2^J = J_11a,9a_*=12.7, H-11*a*(β)), 2.33 (m, 1H, H-23), 2.24 (br. s., 2H, CH_2_-26), 2.20 (m, 1H, H-23′), 2.00–1.93 (m: 2H, [1.97]-H-17, [1.96]-H-11*e*(α)), 1.92–1.82 (m: 2H, [1.87]-H-16, [1.86]-H-6), 1.82–1.72 (m: 3H, [1.79]-H-5, [1.77]-H-22, [1.76]-H-8), 1.70–1.60 (m: 2H, [1.65]-H-15, [1.64]-H-9), 1.53–1.41 (m: 3H, [1.50]-H-4, [1.47]-H-1*, [1.44]-H-7), 1.38–1.14 (m: 9H, [1.36]-H-1′*, [1.32]-H-22′, [1.31]-H-14, [1.31]-H-2*, [1.28]-H-15′, [1.27]-H-16′, [1.22]-H-20, [1.20]-H-2′*, [1.17]-H-6′), 1.10(m, 1H, H-4′), 1.00 (s, 3H, CH_3_-19), 0.97 (m, 1H, H-7′), 0.97 (s, 3H, CH_3_-18), 0.79 (d, 3H, *J_21,20_* = 6.6, CH_3_-21). ^13^C-NMR (CDCl_3_): *δ* = 215.08 (s, C-12), 174.44 (s, C-24), 70.51 (s, C-3), 69.52 (t, C-26), 66.89 (t, C-29), 66.89 (t, C-30), 58.75 (d, C-14), 57.39 (s, C-13), 55.94 (t, C-27), 55.94 (t, C-28), 51.27 (q, C-25), 46.30 (d, C-17), 43.47 (d, C-9), 38.27 (t, C-11), 37.43 (d, C-5), 36.83 (t, C-4), 35.43 (d, C-20), 35.40 (s, C-10), 35.33 (d, C-8), 31.37 (t, C-1*), 31.20 (t, C-2*), 31.10 (t, C-23), 30.34 (t, C-22), 27.34 (t, C-16), 26.42 (t, C-6), 25.84 (t, C-7), 24.14 (t, C-15), 22.77 (q, C-19), 18.41 (q, C-21), 11.50 (q, C-18). Signal assignments marked with an asterisk are unreliable.

### 3.3. Cell Cultures and DCA Derivatives

Human duodenal carcinoma HuTu-80 cells, human hepatocellular carcinoma HepG2 cells, human cervical carcinoma KB-3-1 cells, human lung carcinoma A549 cells were obtained from the Russian Cell Culture Collection (Institute of Cytology, RAS, St. Petersburg, Russia). Non-transformed human fibroblasts hFF3 were kindly provided by Dr. Olga Koval (Institute of Chemical Biology and Fundamental Medicine, SB RAS, Novosibirsk, Russia). RAW264.7 macrophages were kindly provided by Prof. Dr. Dmitry Kuprash (Engelhardt Institute of Molecular Biology, RAS, Moscow, Russia). The cells were cultured in DMEM (Sigma-Aldrich Inc., St. Louis, MO, USA) supplemented with 10% (*v/v*) heat-inactivated fetal bovine serum (FBS) (BioloT, St. Petersburg, Russia), 100 U/mL penicillin (ICN Biomedicals Inc., Costa Mesa, CA, USA), 100 µg/mL streptomycin and 0.25 µg/mL amphotericin; additionally, in the case of RAW264.7 cells, concentration of glucose in the medium was increased up to 4.5 mg/mL. The cells were incubated at 37 °C in 5% CO_2_ and 95% air (hereafter referred to as standard conditions). DCA derivatives were dissolved in DMSO to yield stock solutions of 10 mmol/L and stored at −20 °C prior to use.

### 3.4. Evaluation of Cytotoxicity of DCA Derivatives by MTT Assay

Cells were seeded in triplicate in 96-well plates at a density of 5 × 10^3^ (HuTu-80, HepG2, KB-3-1, hFF3), 10^4^ (A549) or 5 × 10^4^ (RAW264.7) cells per well. Cells were incubated under standard conditions for 24 h and then they were treated with varying doses of investigated derivatives for subsequent 24 h. Then 10 µL of MTT solution (5 mg/mL) were added to each wells and cells were incubated for an additional 2 h under standard conditions. The crystals of formazan forming within live cells were solubilized with DMSO and the absorbance was measured in a Multiscan RC plate reader (Thermo LabSystems, Helsinki, Finland) at a test and reference wavelengths of 570 nm and 620 nm, respectively. The concentrations of compounds caused the decrease optical density at 570 nm to 50% of A_570_ of control non-treated cells (IC_50_) were calculated by extrapolation of dose-response curves. To perform SAR analysis, hierarchical clustering of compounds based on their IC_50_ values was fulfilled with Euclidean distance by using the Morpheus tool (https://software.broadinstitute.org/morpheus/), a platform designed for visualization and analysis of data in a matrix format. Heatmap, containing compounds’ SI values, were constructed by using the Excel 2010 software (Microsoft, Redmond, WA, USA). In order to reveal whether ROS participate in the induction of cell death by compound **9**, HuTu-80 cells cultured in 96-well plate were pretreated by NAC at 2 mM for 1 h. Thereafter, the medium was replaced by fresh medium with compound **9** at 8 µM and the cells were incubated with subsequent 24 h under standard conditions. After this time period, the curves of cytotoxicity of compound **9** in the presence or absence of NAC were compared.

### 3.5. Inhibition of NO Production by Stimulated Macrophages

RAW264.7 cells were seeded in triplicate in 96-well plates at a density of 5 × 10^4^ cells/well and incubated under standard conditions for 24 h. The cells were treated with 20 ng/mL of murine recombinant IFNγ (GIBCO, Gaithersburg, MD, USA), with or without (control) compounds at various concentrations for 24 h. The level of NO production was evaluated by determining the nitrite concentration in culture medium with a Griess Reagent System (Promega, Madison, WI, USA). The IC_50_^NO^ value was calculated as the derivative concentration required to decrease NO production to 50% of the non-treated control.

### 3.6. In Silico ADME Prediction

Theoretical in silico evaluation of ADME properties of compounds was performed using the SwissADME tool (http://www.swissadme.ch) [51]. In this study, we focused on the descriptors, taking into consideration the Lipinski’s Rule of Five, notably molecular weight (MW), Moriguchi octanol-water partition coefficient Log P (mLogP), hydrogen bond acceptors (HBA) and donors (HBD) [28].

### 3.7. Annexin V FITC/PI Apoptosis Detection

HuTu-80 cells were seeded in 6-well plates at a density of 5 × 10^5^ cells per well and incubated for 24 h under standard conditions. Then the medium was replaced with fresh medium containing compound **9** at 8 µM. In order to determine induction of apoptosis, cells were harvested at 6, 18 and 24 h post treatment. Cells were detached with trypsin, washed with cold PBS and then resuspended gently in binding buffer at concentration of 10^6^ cells/mL. Thereafter, cells were stained with fluorescein isothiocyanate (FITC)-conjugated Annexin V and PI (Annexin V-FITC Apoptosis Detection Kit, Millipore, Bedford, MA, USA) for 15 min in the dark and subsequently analyzed using NovoCyte Flow Cytometer (ACEA Biosciences Inc., San Diego, CA, USA). For each sample, 10.000 events were acquired.

### 3.8. Cell Cycle Assay

To determine the effect of compound **9** on the cell cycle, HuTu-80 cells were seeded in 6-well plates at a density of 5 × 10^5^ cells/well for 24 h. After incubation, cells were treated with compound **9** at 8 µM for 6, 18 or 24 h. Thereafter, cells were collected by trypsinization, washed with cold PBS and fixed in 70% ice-cold ethanol for 30 min at 4 °C. Fixed cells were pelleted, stained with PI (1 mg/mL) for 15 min at 37 °C in the dark and analyzed using NovoCyte Flow Cytometer (ACEA Biosciences Inc.). Ten thousand events were recorded for each sample.

### 3.9. Determination of Mitochondrial Membrane Potential (∆ψ_M_)

The mitochondrial-specific cationic dye JC-1 (Molecular Probes, Invitrogen, Carlsbad, CA, USA) accumulating in the mitochondria by potential-dependent manner was used to detect the effect of compound **9** on ∆ψ_M_. Briefly, HuTu-80 cells were seeded in 6-well plates at a density of 5 × 10^5^ cells/well for 24 h. Then cells were treated by compound **9** at 8 µM for 6, 18 or 24 h, collected by trypsinization, washed with PBS, and JC-1 (5 µg/mL) was added to the cells. After 30 min of incubation at 37 °C, the cells were centrifuged, washed with PBS and analyzed using NovoCyte Flow Cytometer (ACEA Biosciences Inc.).

### 3.10. Determination ofCcaspase-3/-7 Activity

The caspase-3/-7 activity in HuTu-80 cells was assessed by using Caspase-Glo^®^ 3/7 Assay kit (Promega, Madison, WI, USA) according to the manufacturer’s instructions. Briefly, the cells were seeded in white-walled 96-well plates at a density of 10^4^ cells/well for 24. After incubation, cells were treated with compound **9** at 8 µM for 6, 18 or 24 h and then 100 µL of Caspase-Glo^®^ 3/7 reagent was added to each well. The plate was carefully shaken for 30 sec on a plate mixer and afterwards incubated for 30 min at room temperature in the dark. The luminescence was detected using CLARIOstar plate reader (BMG Labtech, Ortenberg, Germany).

### 3.11. Autophagy Detection

HuTu-80 cells were seeded in 6-well plates at a density of 5 × 10^5^ cells per well and incubated for 24 h under standard conditions. Then the cells were treated by compound **9** at 8 µM for 24 h, collected by trypsinization, washed with PBS, suspended with 50 µM of MDC at 37 °C for 15 min and analyzed using NovoCyte Flow Cytometer (ACEA Biosciences Inc.). In order to evaluate the type of autophagy (cytoprotective or cytodestructive), HuTu-80 cells were seeded in 96-well plate at a density of 5 × 10^3^ cells/well for 24 h followed by co-treatment with compound **9** at 8 µM and chloroquine, an autophagy inhibitor, at 50 µM for 24 h. After incubation time, the obtained curves of cytotoxicity of compound **9** with the absence or the presence of chloroquine were compared.

### 3.12. Molecular Docking

Docking of compound **9** and VDR was performed using Autodock Vina (The Scripps Research Institute, La Jolla, CA, USA) [52]. The three-dimensional structure of human VDR co-crystalized with LCA propionate (PDB ID: 3W5T) was obtained from the protein data bank (https://www.rcsb.org). Further, the extraction of co-crystalled ligand from the PDB file of the protein, addition of polar hydrogen and Gasteiger charges into protein structure were performed by the AutoDockTools v.1.5.7 (The Scripps Research Institute, La Jolla, CA, USA). The 2D structure of compound **9** was converted to 3D and its geometry was optimized with the universal force field (UFF) using Chem3D (CambridgeSoft, Cambridge, MA, USA) and Avogadro v. 1.2.0 (University of Pittsburgh, Pittsburgh, PA, USA), respectively. All rotatable bonds within the ligand were allowed to rotate freely. The docking grid box sizes were set to 40 × 38 × 36 Å centered on the positions of the ligand (63.63 × 15.31 × (−4.51) Å). Finally, the conformation with the most favorable free energy of binding was selected to analyze the interaction between compound **9** and protein. The results were imported and analyzed using Discovery Studio Visualizer v.17.2.0 (Dassault Systèmes Biovia Corp., San Diego, CA, USA). The 2D plot of the protein-ligand interactions was analyzed using LigPlot+ v.1.4.5 (European Bioinformatics Institute, Cambridge, UK).

## 4. Conclusions

In summary, a series of novel DCA derivatives **2**–**10**, bearing aliphatic diamine and aminoalcohol as well as morpholine moieties at the C3 position, were synthesized and tested for cytotoxicity against four human cancer cell lines, murine macrophages and non-malignant human fibroblasts in vitro as well as for their ability to suppress NO production by macrophages. We showed that all introduced amine-containing substituents significantly increased the cytotoxicity of the novel compounds in comparison with the parent molecule DCA and did not impart inhibitory activity against macrophage-mediated NO synthesis. The evaluated compounds were shown to display pronounced selectivity of anti-proliferative effect for enterohepatic tumor cell lines HuTu-80 and HepG2, which markedly increased when tertiary amino groups in the side moieties of derivatives (compounds **2**/**5** and **3**/**6**) were replaced by hydroxyl groups (compounds **8** and **9**). Compound **9**, bearing an aminopropanol substituent at the C3 position, was identified as a lead compound, displaying the highest Selectivity Index in the tested series for the most susceptible HuTu-80 cells and good drug-likeness parameters. Mechanistic studies of compound **9** in HuTu-80 cells revealed that this derivative induced ROS-dependent cell death by activation of intrinsic caspase-dependent apoptosis and cytodestructive autophagy. The results from molecular docking simulations showed that VDR can be considered as a primary intracellular target of compound **9**. Taken together, our findings provide some new suggestions for the design of novel antitumor agents based on the BA scaffold and a basis for the better understanding of the mechanism of action of BA derivatives.

## Figures and Tables

**Table 1:** Cytotoxicity and anti-NO activity of novel DCA derivatives.

Compound	IC50 (µM)						IC50NO (µM)
	Duodenum	Liver	Cervix	Lung	Macrophage	Fibroblast	
	HuTu-80	HepG2	KB3-1	A549	RAW264.7	hFF3	
1 *	17.0 ± 1.6 *	43.5 ± 1.9 *	27.9 ± 2.5 *	ND	>100	53.9 ± 3.3 *	16.4 ± 0.9
2 *	7.1 ± 0.6 *	8.0 ± 0.9 *	26.2 ± 0.8 *	36 ± 0.7 *	21.7 ± 0.87	25.4 ± 2.2 *	>10
3 *	3.0 ± 0.4 *	4.0 ± 0.2 *	5.9 ± 0.5 *	6.8 ± 0.1 *	6.7 ± 0.4	6.5 ± 0.5 *	>10
4 *	4.7 ± 0.2 *	3.6 ± 0.7 *	23.5 ± 1.7 *	21.6 ± 2.5 *	20.4 ± 0.4	32.5 ± 1.4 *	>10
5 *	3.3 ± 0.1 *	4.3 ± 0.3 *	21.6 ± 3.4 *	25.5 ± 0.8 *	20.9 ± 2.1	7.4 ± 1.0 *	>10
6 *	1.0 ± 0.6 *	3.7 ± 0.17 *	6.7 ± 0.6 *	6.8 ± 0.3 *	6.7 ± 0.5	6.2 ± 0.4 *	>10
7 *	8.3 ± 1.2 *	3.8 ± 0.3 *	28.6 ± 0.5 *	23.5 ± 3.0 *	37.8 ± 1.7	17.4 ± 1.6 *	>10
**8**	4.4 ± 0.34	5.8 ± 1.3	17.7 ± 2.8	29.6 ± 2.5	23.4 ± 1.2	34.8 ± 1.0	>10
**9**	4.0 ± 0.5	7.3 ± 0.6	22.8 ± 1.8	28.98 ± 3.5	19.3 ± 3.7	33.3 ± 1.2	>10
**10**	10 ± 0.3	7.5 ± 1.2	8.4 ± 0.5	>50	>50	>50	>10
11 *	>100 *	25.7 ± 3.6 *	>100 *	ND	>100	>100 *	50.9 ± 5.9
DCA *	82.9 ± 1.9 *	>100 *	>100 *	ND	>100	>100 *	83.6 ± 1.6

The IC_50_ values were determined as the concentration of derivatives causing the death of 50% of cells (24 h). The IC_50_^NO^ was calculated as the concentration of the derivative leaded to 50% reduction in NO synthesis by IFNγ-activated RAW264.7 cells in comparison with non-treated IFN γ-stimulated control (24 h). ND—not determined. * Asterisk shows data from our previous works [21,22,23].

**Table 2:** In silico drug-like properties of compounds **1**–**11** according to Lipinski’s Rule of Five.

Compound	MW (Da) ^a^	mLogP ^b^	HBA ^c^	HBD ^d^	Lipinski Violation
Rule	≤500	≤5	≤10	≤5	0
**1**	477.68	2.94	6	3	0
**2**	504.74	3.33	6	2	1
**3**	518.77	3.52	6	2	1
**4**	516.76	3.52	6	1	1
**5**	532.8	3.71	6	2	1
**6**	546.82	3.89	6	2	1
**7**	530.78	3.71	6	1	1
8*	477.68	2.94	6	3	0
9*	491.7	3.14	6	3	0
**10**	503.71	3.33	6	1	1
**11**	404.58	3.98	4	1	0

Abbreviations: ^a^ Molecular weight; ^b^ Moriguchi logarithm of the compound partition coefficient between n-octanol and water; ^c^ Number of hydrogen bond acceptors; ^d^ Number of hydrogen bond donors. Asterisk shows the lead compounds chosen for further evaluation of their mechanism of action.

## Supplementary Materials

The ^1^H- and ^13^C-NMR spectra of the synthesized compounds are available online.
