# Supplementary material for: Novel Derivatives of Deoxycholic Acid Bearing Linear Aliphatic Diamine and Aminoalcohol Moieties and their Cyclic Analogs at the C3 Position: Synthesis and Evaluation of Their In Vitro Antitumor Potential
Source: Molecules. 2019 Jul 21;24(14):2644. doi: 10.3390/molecules24142644 (PMC6681416; doi:10.3390/molecules24142644)
Supplement: Supplementary file 1 [file molecules-24-02644-s001.zip › Markov et al. Molecules_SUPPORTING INFORMATION.docx]

**ELECTRONIC SUPPORTING INFORMATION**

**1. NMR ^1^H and ^13^C spectra of the synthesized compounds**

Spectra of Compound **1**, ^1^H NMR, 600MHz, CDCl_3_

Spectra of Compound **1**,^13^C NMR, JMOD, 150MHz, CDCl_3_

High resolution mass spectra of compound **1**, Tsource=230°C

Calculated m/z=416.2921 (C_26_H_40_O_4_)^+^

Found m/z=416.2922

Spectra of Compound **2**, ^1^H NMR, 600MHz, CDCl_3_

Spectra of Compound **2**,^13^C NMR, JMOD, 150MHz, CDCl_3_

High resolution mass spectra of compound **2**, Tsource=80°C, Tprobe=240°C

Calculated m/z = 504.3922 (C_30_H_52_O_4_N_2_)^+^

Found m/z = 503.3837

Spectra of Compound **3**, ^1^H NMR, 600MHz, CDCl_3_

Spectra of Compound **3**,^13^C NMR, JMOD, 150MHz, CDCl_3_

High resolution mass spectra of compound **3**, Tsource=80°C, Tprobe=200°C

Spectrum in the region of molecular ion

Calculated m/z = 518.4078 (C_31_H_54_O_4_N_2_)^+^

Found m/z = 518.4068

Spectra of Compound **4**, ^1^H NMR, 600MHz, CDCl_3_

Spectra of Compound **4**,^13^C NMR, JMOD, 150MHz, CDCl_3_

High resolution mass spectra of compound **4**, Tsource=70°C, Tprobe=220°C

Spectrum in the region of molecular ion

Calculated m/z=516.3922 (C_31_**H_52_**O_4_N_2_)^+^

Found m/z=515.3847

Calculated m/z=515.3843 (C_31_**H_51_**O_4_N_2_)^+^

Spectra of Compound **5**, ^1^H NMR, 600MHz, CDCl_3_

Spectra of Compound **5**,^13^C NMR, JMOD, 150MHz, CDCl_3_

High resolution mass spectra of compound **5**, Tsource=80°C, Tprobe=200°C

Spectrum in the region of molecular ion

Calculated m/z=532.4235 (C_32_H_56_O_4_N_2_)^+^ **(M) ^+^**

Found m/z=531.4151 **(M–H) ^+^**

Calculated m/z=531.4156 (C_32_H_55_O_4_N_2_)^+^ **(M–H) ^+^**

Found m/z=514.4130 **(M–H_2_O) ^+^**

Calculated m/z=514.4129 (C_32_H_54_O_3_N_2_)^+^ **(M–H_2_O) ^+^**

Spectra of Compound **6**, ^1^H NMR, 600MHz, CDCl_3_

Spectra of Compound **6**,^13^C NMR, JMOD, 150MHz, CDCl_3_

High resolution mass spectra of compound **6**, Tsource=80°C, Tprobe=200°C

Spectrum in the region of molecular ion

Calculated m/z=546.4391 (C_33_H_58_O_4_N_2_)^+^ **(M)^+^**

Found m/z=545.4294 **(M–H)^+^**

Calculated m/z=545.4313 (C_33_H_57_O_4_N_2_)^+^ **(M–H)^+^**

Found m/z=528.4289 **(M–H_2_O)^+^**

Calculated m/z=528.4286 (C_33_H_56_O_3_N_2_)^+^ **(M–H_2_O)^+^**

Spectra of Compound **7**, ^1^H NMR, 600MHz, CDCl_3_

Spectra of Compound **7**,^13^C NMR, JMOD, 150MHz, CDCl_3_

High resolution mass spectra of compound **7**, Tsource=80°C, Tprobe=260°C

Spectrum in the region of molecular ion

Calculated m/z=530.4078 (C_32_H_54_O_4_N_2_)^+^

Found m/z=529.3995

Found m/z=530.4070

Calculated m/z=529.4000 (C_32_H_53_O_4_N_2_)^+^

Spectra of Compound **8**, ^1^H NMR, 600MHz, DMSO-d6

Spectra of Compound **8**,^13^C NMR, JMOD, 150MHz, DMSO-d6

High resolution mass spectra of compound **8**, Tsource=50°C, Tprobe=200°C

Calculated m/z=477.3449 (C_28_H_47_O_5_N_1_)^+^

Found m/z=477.3456

Spectra of Compound **9**, ^1^H NMR, 600MHz, CDCl_3_

Spectra of Compound **9**,^13^C NMR, JMOD, 150MHz, CDCl_3_

High resolution mass spectra of compound **9**, Tsource=60°C, Tprobe=250°C

Calculated m/z=491.3605 (C_29_H_49_O_5_N_1_)^+^

Found m/z=491.3607

Spectra of Compound **10**, ^1^H NMR, 600MHz, CDCl_3_

Spectra of Compound **10**,^13^C NMR, JMOD, 150MHz, CDCl_3_

High resolution mass spectra of compound **10**, Tsource=70°C, Tprobe=250°C

Calculated m/z=503.3605 (C_30_H_49_O_5_N)^+^

Found m/z=503.3596
